# Lipid Diffusion in Supported Lipid Bilayers: A Comparison between Line-Scanning Fluorescence Correlation Spectroscopy and Single-Particle Tracking

**DOI:** 10.3390/membranes5040702

**Published:** 2015-11-13

**Authors:** Markus Rose, Nehad Hirmiz, Jose M. Moran-Mirabal, Cécile Fradin

**Affiliations:** 1 Department of Physics and Astronomy, McMaster University, Hamilton, ON L8S 4M1, Canada; E-Mails: rosemm2@mcmaster.ca (M.R.); hirmizn@mcmaster.ca (N.H.); 2 Department of Chemistry and Chemical Biology, McMaster University, Hamilton, ON L8S 4L8, Canada; 3 Department of Biochemistry and Biomedical Sciences, McMaster University, Hamilton, ON L8N 3Z5, Canada

**Keywords:** lipid, bilayer, membrane, diffusion, fluorescence, confocal microscopy, total internal reflection fluorescence microscopy (TIRF), FCS, SPT

## Abstract

Diffusion in lipid membranes is an essential component of many cellular process and fluorescence a method of choice to study membrane dynamics. The goal of this work was to directly compare two common fluorescence methods, line-scanning fluorescence correlation spectroscopy and single-particle tracking, to observe the diffusion of a fluorescent lipophilic dye, DiD, in a complex five-component mitochondria-like solid-supported lipid bilayer. We measured diffusion coefficients of *D*_FCS_ ~ 3 μm^2^ · s^−1^ and *D*_SPT_ ~ 2 μm^2^ · s^−1^, respectively. These comparable, yet statistically different values are used to highlight the main message of the paper, namely that the two considered methods give access to distinctly different dynamic ranges: *D* ≳ 1 μm^2^ · s^−1^ for FCS and *D* ≲ 5 μm^2^ · s^−1^ for SPT (with standard imaging conditions). In the context of membrane diffusion, this means that FCS allows studying lipid diffusion in fluid membranes, as well as the diffusion of loosely-bound proteins hovering above the membrane. SPT, on the other hand, is ideal to study the motions of membrane-inserted proteins, especially those presenting different conformations, but only allows studying lipid diffusion in relatively viscous membranes, such as supported lipid bilayers and cell membranes.

## 1. Introduction

Fluorescence microscopy is an invaluable tool for the characterization of living systems. Through the development of ever-improved dyes and minimally-invasive tagging methods, it has become possible to image a large array of biological samples, including live cells [1,2,3]. A large toolbox of fluorescence imaging techniques is available, from ensemble methods, such as fluorescence recovery after photobleaching (FRAP) and fluorescence correlation spectroscopy (FCS) [4], to single-particle detection techniques [5] and super-resolution imaging, like stochastic optical reconstruction microscopy (STORM) and stimulated emission depletion (STED) microscopy [6]. Ensemble and single-particle methods notably differ in the way the fluorescence data are processed. In ensemble methods, information about the system is extracted using a signal that is the sum of the signals generated by many individual molecules and often obtained in response to an external perturbation. For FRAP, for example, the external stimulus is a photobleaching step, while for FCS, it is thermal perturbations. In single-particle methods, on the other hand, an analysis software is used to identify and extract the fluorescence signal generated by individual particles. While the ensemble methods can more easily achieve large signal-to-noise ratios and are appropriate for a wide range of fluorophore concentrations, single-particle methods provide a density of information that becomes especially interesting in the case of heterogeneous systems, in which not all particles behave in the same way.

Most cellular processes involve diffusion or transport processes. In particular, the importance of dynamics in biological membranes, including lipid and protein diffusion and membrane-protein interactions, cannot be overstated [7,8]. We thus need fluorescence techniques dedicated to studying dynamics in membranes. Several of our previous works, devoted to the diffusion of proteins in either cells [9] or reconstituted membranes [10], have illustrated the often neglected fact that different techniques have quite specific dynamic ranges that need to be carefully considered. In this paper, we propose to directly compare two such techniques, one ensemble average technique (line-scanning FCS) and one single-particle technique (single-particle tracking). Line-scanning FCS [11,12,13,14] is in essence a simplified version of raster image correlation spectroscopy (RICS) [15,16]. In this approach, the data are acquired with a laser-scanning confocal microscope. The intensities of every pair of pixels in each line are then correlated to detect characteristic correlation decay times corresponding to dynamic processes, for example the diffusion of fluorescent particles through the detection volume. Correlation spectroscopy techniques have been used on many occasions for the study of lipid diffusion [17,18,19,20,21,22,23]. On the other hand, single-particle tracking (SPT) [5,24] is usually implemented on data acquired with a total internal reflection fluorescence (TIRF) microscope, where all pixels in each image are acquired simultaneously. The images are then analysed to precisely locate each fluorescent molecule within the image, and molecular tracks are built by linking individual molecule positions across frames. SPT has also been commonly used in lipid diffusion studies [5,24,25,26,27,28].

For a fair side-by-side comparison of these two techniques, we applied them to measure the diffusion coefficient of a standard lipophilic dye, DiD (1,1′-dioctadecyl-3,3,3′,3′-tetramethylindocarbocyanine), in the exact same membrane. We chose to use a five-component lipid bilayer, with a composition reflecting that of the outer mitochondrial membrane, which we had previously employed as a model mitochondria-like membrane [10,29,30,31,32]. Although its lipid composition partially reflects the complexity of actual cellular membranes, it remains an easily-prepared model system, which has already been well-characterized both in terms of structure [33] and diffusion properties [10]. Interestingly, proteins inserted in this membrane were shown to exhibit different modes of motion, from immobile to fast two-dimensional diffusion [10]. Thus, we used this composition in the hope of observing heterogeneous motions of the lipid molecules, and to see how both methods would fare in distinguishing populations with different mobilities. Solid-supported lipid bilayers with a very small concentration of DiD were prepared and successively imaged by confocal microscopy and TIRF microscopy. The confocal images were then used to generate line autocorrelation functions, while the TIRF movies were used to implement single-particle tracking. Assuming simple diffusion, we used both sets of data to extract the diffusion coefficient of mobile DiD molecules in the membrane and show that, for this particular well-chosen system, both methods return a similar value. However, when comparing them, we highlight that they have very different dynamic ranges. We place this in the context of membrane studies and advise on which method should be used for which system.

## 2. Results

### 2.1. Line-Scanning Fluorescence Correlation Spectroscopy

The information contained in a single confocal image exceeds the simple location of fluorescent molecules. Each pixel represents the intensity, or number of photons, collected from the small confocal illumination volume when placed at that position over a short period of time (the pixel dwell time, *δ*). Fluctuations in intensity are related to both fluctuations in fluorophore concentration in space and time and fluctuations of fluorescence signal (shot noise). The images are constructed by scanning the laser beam used for excitation and, thus, scanning the confocal volume in the sample, pixel by pixel and line by line. Because each pixel is acquired at a different time, a confocal image contains information about the motion of the imaged fluorescent molecules [15].

**Figure 1:**
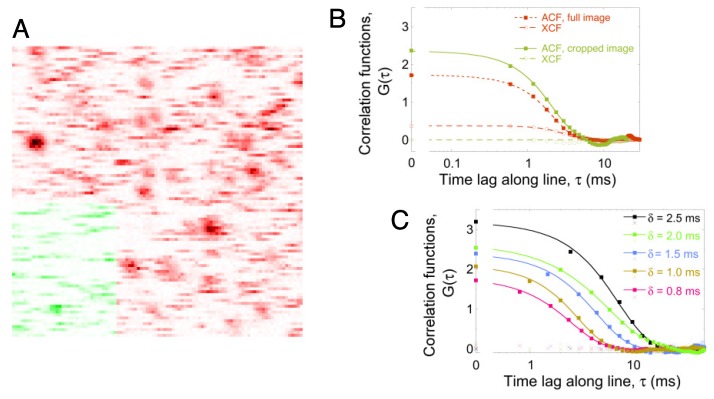
(**A**) Confocal image of a mitochondria-like lipid bilayer containing 0.0025 wt% DiD. Both streaks (corresponding to diffusing DiD molecules) and spots (corresponding to immobile or very slow DiD molecules) can be seen in this image. The image has been inverted for clarity. The highlighted green part seems to contain mostly streaks and was cropped and used for separate analysis. Image size: 10 μm × 10 μm; pixel size: *d* = 100 nm; pixel dwell time: *δ* = 0.6 ms. (**B**) Average line autocorrelation functions (filled squares) and line cross-correlation functions (empty circles) computed for either the full image (red symbols) or the cropped portion of the image highlighted in green (green symbols) in (A). The data have been fit with Equation (1) (continuous line), Equation (2) (dashed lines) or Equation (3) (dotted line). (**C**) Line autocorrelation functions (filled squares) and line cross-correlation functions (crosses) obtained for different images of the sample shown in (A), imaged at different pixel dwell times and cropped to include only mobile particles. The line autocorrelation functions have been fit with Equation (1) (continuous line).

Figure 1A shows a typical confocal image of a solid-supported lipid bilayer containing a very small concentration (0.0025 wt%) of fluorescent molecules (here, the lipophilic fluorophore DiD). In practice, several confocal images were recorded in succession, each spaced by a small distance (0.5 to 1 μm) along the optical axis. This was done to ensure that at least one image in the stack was acquired with the lipid bilayer in focus (generally easily identifiable as the one with the largest average intensity). Acquiring a stack also allowed identifying areas of the image with or without immobile particles, by calculating an average line cross-correlation function, as explained below. The image in Figure 1A shows the lipid bilayer in sharp focus. In this case, because the fluorophore concentration was very low, individual fluorescent molecules can be observed. In addition, because the distance between consecutive pixels (*d* = 100 nm) was chosen to be smaller than the radius of the confocal volume (*ω*_0_ ≃ 350 nm), each fluorescent molecules can be observed for several consecutive pixels along a line. The signature of each molecule is different depending on its mobility [10]. Immobile fluorophores are detected over several consecutive lines and appear as Gaussian spots (which are images of the confocal detection volume and, therefore, have a radius ~ *ω*_0_). On the other hand, diffusing fluorophores detected in one line have cleared the area before the next line is imaged and appear as horizontal streaks. Both spots and streaks are visible in Figure 1A, indicating the presence of both immobile and diffusing DiD molecules. The green area of the image highlights an area of the image in which no immobile particles are in evidence.

The extraction of the dynamic information contained in confocal images can be done using image correlation spectroscopy (ICS), in which pixels in successive images are correlated [34,35], or using raster image correlation spectroscopy (RICS), in which pixels within the same image are correlated [15,16]. However, ICS does not allow studying fast dynamics, such as lipid diffusion, while RICS does. With RICS, a two-dimensional spatio-temporal autocorrelation function (ACF) of the image is calculated, then fit with a model function assuming a particular type of motion, for example simple diffusion. This type of analysis becomes rapidly complicated if there is more than one fluorescent species in the image. For this work, we used a simpler one-dimensional version of RICS, namely line-scanning FCS, where the ACF is only computed in the horizontal direction, along the direction of scanning. This was done using a dedicated ImageJ plugin, described in detail in [36]. The result is a one-dimensional ACF, *G*(*τ*), each point of which represents an average of the correlation between all pairs of points separated by a certain distance (and thus, by a certain lag time, *τ*) along lines in the image. One particularity of our algorithm is that it includes a procedure to remove the shot-noise contribution from the first point in the correlation function (*τ* = 0), in which case *G*(0) = 1/〈*N*〉, where *N* is the number of fluorescent particles in the detection volume (see the Methods Section for details). Thus, *G*(0) can immediately be used to calculate fluorophore concentration. The line ACF calculated for the image in Figure 1A is shown in Figure 1B, where the logarithmic axis has been interrupted in order to show the value of *G*(0) and to highlight its importance. For the studied samples, the amplitude of the ACF was *G*(0) ~ 2, corresponding to a probe surface concentration of [*C*] ~ 1.3 molecule μm^−2^.

In addition to the line ACF, a line cross-correlation function (CCF), *G_X_*(*τ*), was also calculated, by correlating the intensity of pixels in the image of interest with those of the pixels in the next image in the stack. A non-zero CCF is expected only if some of the particles detected in the first image are still in the same position in the second image, *i.e.*, in the presence of immobile particles. In that case, *G_X_*(0) = 1/〈*N*_immobile_〉 should be related to the fraction of immobile particles in the detection volume. Since the image in Figure 1A does contain Gaussian spots indicating the presence of immobile particles, we do expect a non-zero CCF when correlating that image to the next image in the stack. Indeed, a distinctly non-zero CCF is observed for this image (Figure 1B). On the other hand, when selecting an area of the image with only streaks and no Gaussian spots (such as the green area highlighted in Figure 1A), we expect a zero-amplitude CCF. Indeed, when calculating the CCF for a cropped image containing only that area, we obtained a zero-amplitude CCF, confirming that no immobile particles are present in this portion of the image (Figure 1B). Calculating the CCF thus represents a quick and unbiased way of assessing whether an image or a portion of an image may contain immobile particles. This, in turn, allows one to decide which model to use when fitting the ACF to extract the diffusion coefficient of the imaged particles, as discussed below.

In order to retrieve the diffusion coefficient of the particles, one needs to fit the ACF with the proper model. In the case of a sample containing a single species of particles diffusing with a diffusion coefficient *D*, the line ACF should take the form captured in Equation (1) [37]. Two characteristic times appear in that equation. The first, the characteristic scan time, *τ_S_* = *ω*_0_*δ*/*d*, is related to the time an immobile particle would spend in the scanning detection volume and, therefore, directly depends on the scanning speed, *d*/*δ*. The second, the characteristic diffusion time, $\tau_{D}=\omega_{0}^{2}/\left(4D\right)$, is related to the time a diffusing molecule would spend in the detection volume if the latter were immobile. Because of the rather small pixel dwell times used here, necessary in order to obtain a good time resolution, we were in a regime where *τ_S_* < *τ_D_*, and therefore, the ACF is dominated by *τ_S_*. This can be seen in Figure 1C, where increasing *δ*, and therefore, increasing the value of *τ_S_*, pushes the decay of the ACF towards larger lag times. Equation (1) was used to fit ACFs calculated for images or areas that did not contain immobile particles, *i.e.*, images for which *G_X_*(0) = 0 (as is the case, for example, in Figure 1C). In spite of the dominance by *τ_S_*, the shape of the ACF is sufficiently modulated by *τ_D_* for the fit to return a reasonably precise value of this parameter.

In the presence of immobile particles, a second term needs to be added to the model ACF used for fitting (to account for these immobile particles), giving Equation (3). We note that using Equation (3), which has one more fitting parameter than Equation (1) (related to the relative fraction of immobile particles), gives fit parameter values that are less precise than when using Equation (1); this is is the price to pay in order to disentangle the contribution of the immobile particles in the ACF. Using a cropped area without mobile particles, as illustrated in Figure 1, is therefore interesting. However, cropping results in a loss of statistics when calculating the ACF, and this is especially visible at long lag times (compare both ACFs in Figure 1B, where the ACF obtained from the cropped image starts having oscillations at a long lag time).

**Figure 2:**
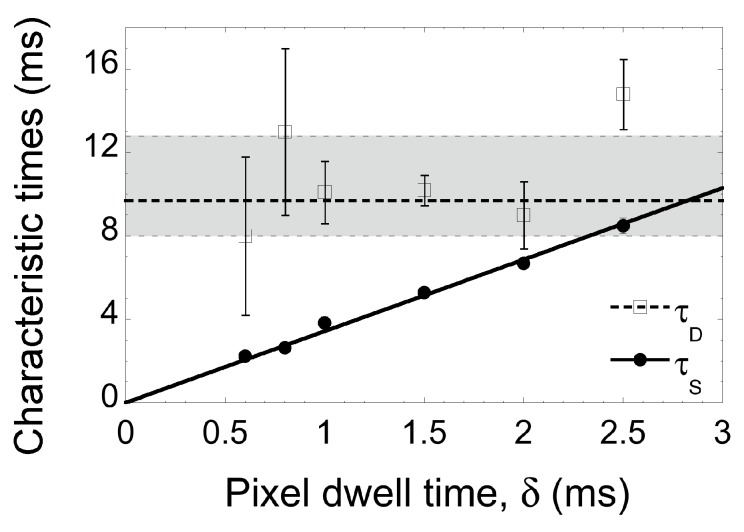
Values of the characteristic diffusion time (*τ_D_*) and characteristic scanning time (*τ_S_*) obtained for images acquired at different dwell times (mean ± SEM). As expected, *τ_S_* increases linearly with *δ*, and a linear fit of the data (solid line) gives *ω*_0_ = 345 nm. Furthermore, as expected, the diffusion time is constant. The bold dashed line indicates the value of the 50th percentile (median) of *τ_D_* values and the light dashed lines those of its 30th and 70th percentiles. The median value, *τ_D_* = 9.7 ms, corresponds to a diffusion coefficient $D=\omega_{0}^{2}/\left(4\tau_{D}\right)$ = 3.1 μm^2^ · s^−1^.

To measure the diffusion coefficient of DiD in the supported mitochondria-like lipid bilayer chosen for this study, we analysed a number of confocal stacks acquired on the same sample with different pixel dwell times. For each stack, the brightest image was selected. Analysis of the ACF calculated for the full image was first performed using Equation (3) (if *G_X_*(0) ≠ 0), or Equation (1) (if *G_X_*(0) = 0). Then, regions of the sample where no immobile particles (spots) were visible were selected and the corresponding ACFs analysed with Equation (1) (after we verified that *G_X_*(0) = 0). Values of *τ_D_* and *τ_S_* obtained for different images obtained in the same conditions (same *δ*) and by both methods were averaged together and are shown in Figure 2. The characteristic scan time is obtained with great precision, since it dominates the correlation function (*τ_S_* < *τ_D_*). As expected, it depends linearly on the pixel dwell time *δ*. Using a linear fit on *τ_S_*(*δ*) returns a value for *ω*_0_, and we find *ω*_0_ ≃ 345 nm. Line-scanning FCS therefore provides a direct way of calibrating the detection volume that does not rely on the knowledge of the diffusion coefficient of a calibration dye. The characteristic diffusion time is measured with a lower precision, but it still appears that, as expected, *τ_D_* does not depend on *δ*.

We found that using full images (and either Equation (3) or Equation (1) depending on the value of *G_X_*(0)) or using cropped images without immobile particles (and Equation (1)) returned similar average values of *τ_D_* and, therefore, of the diffusion coefficient *D*. In the first case, we obtained *D* = (3.4 ± 0.4) μm^2^ · s^−1^ and in the second case *D* = (3.2 ± 0.4) μm^2^ · s^−1^ (mean ± SEM) (both close to the median value *D* = 3.1 μm^2^ · s^−1^, a good indication that outlier data points do not have too much of an influence on the data).

### 2.2. Single-Particle Tracking

**Figure 3:**
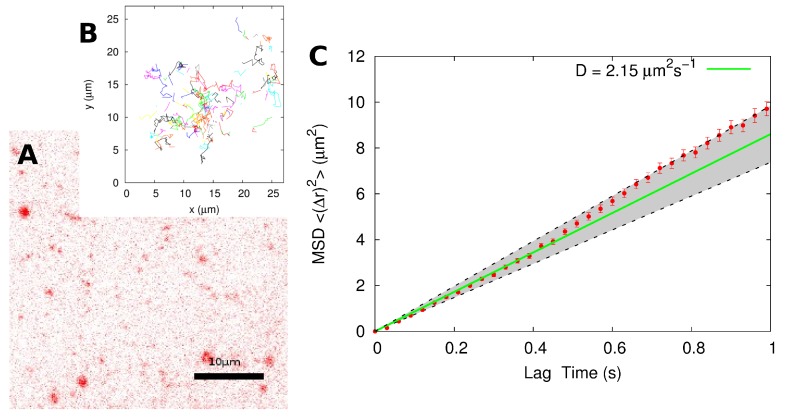
(**A**) Image of a mitochondria-like lipid bilayer imaged with TIRF microscopy. Image size: 41 μm × 41 μm; pixel size: *d* = 160 nm; frame interval: *δ* = 30 ms. (**B**) Tracks generated from a 5-min time-lapse movie. The particle positions are obtained though localization and detection of the particles in each frame of the time lapse movie. After this, the positions are linked from frame to frame to create the tracks. (**C**) Mean-squared displacement 〈(Δ*r*)^2^〉 obtained for the combined track. The error bars represent the standard error of the mean. The diffusion coefficient is obtained through a linear fit via the equation 〈(Δ*r*)^2^〉 = 4*Dτ*. Fitting over regions of different lag times ranging from *τ* = 0.1 s to 0.9 s, we obtained a diffusion coefficient in the range 1.8 μm^2^ · s^−1^ to 2.4 μm^2^ · s^−1^ (shaded region). Using data up until *τ* = 0.5 s gave *D* = (2.2 ± 0.4) μm^2^ · s^−1^.

To directly compare line-scanning FCS and SPT, the same samples that were imaged by confocal microscopy for FCS were then immediately imaged on a separate TIRF microscope, and time-lapse movies were acquired. Figure 3A gives an example of a TIRF image of a sample (DiD immersed in a supported mitochondria-like lipid bilayer, the same as in Figure 1A). An SPT algorithm (described in detail in the Methods Section) was used to generate the tracks of each individual molecule (see Figure 3B). Because we are interested here mainly in the mobile DiD molecules, immobile particles were excluded from the analysis; this can be done in a straightforward way by setting a threshold value for the average step size in a trajectory: all particles with an average step size below a threshold value of 0.05 μm (roughly corresponding to a threshold diffusion coefficient of *D* = 0.08 μm^2^ · s^−1^) were considered immobile and rejected. The remaining tracks correspond to mobile particles and constituted the data from which the diffusion coefficient were extracted in multiple ways.

First, we considered the averaged mean-squared displacement (MSD) obtained from a composite track (Figure 3C). We created the composite track by appending the beginning of one trajectory to the end of another in a random fashion. All together, we appended 130 tracks with a total of 1603 steps. The value of the diffusion coefficient obtained by the linear fit of the MSD (using Equation (4)) varies slightly depending on the region chosen for the fit. Using data up to the lag time *τ* = 0.5 s (which was chosen because it is the mean single track length for this dataset) results in *D* 2.2 μm^2^ · s^−1^. Between the lag times 0.1 s and 0.9 s, each of which is one standard deviation away from the mean track length, we obtain a range of diffusion coefficients from 1.8 μm^2^ · s^−1^ to 2.4 μm^2^ · s^−1^ (shaded region in Figure 3C). Thus, the measured diffusion coefficient using this particular analysis method is given as *D* = (2.2 ± 0.4) μm^2^ · s^−1^.

**Figure 4:**
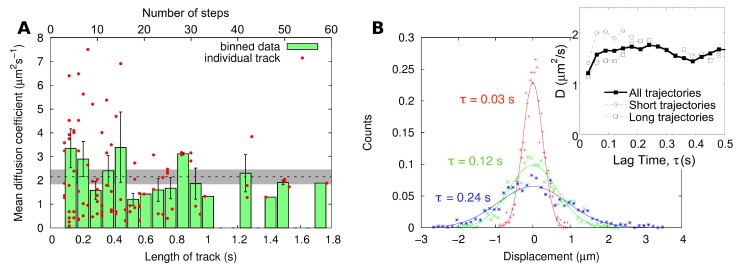
(**A**) A comparison between the length of the acquired track and the determined diffusion coefficient using mean-squared displacement (MSD) analysis of single tracks. The spread of the diffusion coefficient values decreases with track length. The dotted line indicates the value of the diffusion coefficient determined using MSD analysis of the combined track (*D* = 2.2 μm^2^ · s^−1^). (**B**) Distribution of displacements for all trajectories at different lag times (coloured symbols). Each of these distributions is fitted with a normal distribution according to Equation (5) (solid lines). The inset shows the dependence of the value of the diffusion coefficient obtained in this way as a function of lag time, when considering all trajectories (filled symbols) or only short (less than 0.5 s) or long (more than 1 s) trajectories.

When comparing the diffusion coefficients for all of the tracks, separated into different track lengths, no clear correlation can be established, as can be seen in Figure 4A. This shows that the track length does not skew the diffusion coefficient recovered for the particle (a result in agreement with previous observations made for the diffusion of beads in a water and glycerol solution [38]). We do however notice a large spread when it comes to very short tracks (which are more numerous), as predicted by statistical considerations and simulations [39,40] and as experimentally observed [38]. This means that a precise value of the diffusion coefficient cannot be recovered for a single particle, unless a very long track (more than 1 s for the considered sample) is captured, which due to photobleaching only happened here for 7% of the particles. A diffusion coefficient can be estimated for each individual tracked particle, by generating the MSD for that particle and then fitting it with Equation (4). From this distribution of the diffusion coefficient, we obtained a second estimate of the average diffusion coefficient of DiD in the membrane, *D* = (2.4 ± 0.3) μm^2^ · s^−1^ (mean ± SEM).

Taking the analysis one step further, histograms of displacement for different lag times can be generated, as shown in Figure 4B. These distributions of displacements seemed to follow normal distributions, as expected for simple Brownian motion. Fitting these distributions with Equation (5), we obtained for the shortest lag time (*τ* = 0.03 s, corresponding to a single step) a diffusion coefficient of *D* = 1.2 μm^2^ · s^−1^. However, for larger lag times, the retrieved diffusion coefficient approached the value obtained through MSD analysis (Figure 4B, inset). The discrepancy between the value of *D* obtained at the shortest lag time and those obtained for other lag times exists both for short and long trajectories (Figure 4B, inset) and is therefore not due to a bias in which types of particles are detected for only short or long times. Although we do not have a definitive explanation for this discrepancy, it could in principle be due to deviation from simple diffusion. It is, for example, possible that the tracked mobile DiD molecules sometimes get trapped and remain confined for a few steps. This intermittent mobility behaviour was clearly observed for proteins embedded in that same type of lipid bilayer [10]. It might therefore occur for the lipophilic tracer. Analysing distributions of displacements, or the distributions of diffusivities that can be obtained straightforwardly from it, is in principle a good way to bring to light heterogeneous or anomalous diffusion behaviours [41,42]. However, in the present case, the quality of the single-particle data does not allow one to observe that clearly. An alternative explanation for the discrepancy between the value of *D* recovered for the distribution of displacement obtained for a single step and all of the others could be the imperfect localization of the particles. This would disproportionately affect the measurements of short displacements and, therefore, short lag time histograms. The first point of the MSD shown in Figure 3 displays the same atypical behaviour. Averaging over all lag times up to *τ* = 0.5 s, the fit of the distribution of displacement gives us *D* = (1.6 ± 0.2) μm^2^ · s^−1^ (mean ± SD).

## 3. Discussion

Comparing different methods for measuring diffusion in lipid membranes is not trivial since, even for the exact same lipid composition and fluorescent probe; the measured diffusion coefficient depends on many factors, for example temperature [43], solution ionic strength [44], the type of reconstituted membrane used [22] and the type of substrate in the case of supported lipid bilayers [45]. The motivation of this study was therefore to compare two different methods using the exact same sample. Although this had been done before [22], one particularity of the present study is that we utilized a complex five-component lipid bilayer, in which some of the detected particles were immobile, thereby providing a test for the methods used as to whether the immobile particles could be separated from the mobile particles. Another distinctive aspect of our study is that, for each method, we endeavoured to analyse the data in different ways, to see how that would affect the value of the measured diffusion coefficient (yet, we note that our analyses are all based on the assumption, discussed in more detail below, that the studied diffusive process could be assimilated to simple diffusion). We otherwise used a standard reporter dye and standard imaging conditions, in order to be able to draw general conclusions about these methods.

The first chosen method was line-scanning FCS [11,12,13,14,36]. It represents an excellent compromise between the simpler single-point FCS, which is not well adapted to the relatively slow diffusion found in membranes, as it leads to photobleaching, and the more complex RICS, which is advantageous in the case of very heterogeneous samples, such as cell membranes, but not required for the more homogeneous reconstituted lipid bilayers. Line-scanning FCS also allows an accurate calibration of the detection volume (just like circular-scanning FCS [46]), which removes a source of systematic error that has plagued single-point FCS studies and allows a more meaningful comparison with other methods. The second method was SPT, which it is often regarded as the foremost way to study motion in membranes [5,24,28].

A first, and often discussed, point of comparison for these two methods is the available concentration range. SPT relies on a sample preparation leading to a fluorescent probe with a concentration in the single molecule range, whereas FCS methods can be used for a higher and wider concentration range [22,36]. For SPT, one wants less than one fluorescent particle on average within the detection volume (*N* ≪ 1) [47]. For FCS, on the other hand, *N* between 1 and 10 is ideal. The higher concentration limit is set by single particle fluctuations becoming too small compared to the average fluorescence intensity (*i.e.*, the amplitude of the ACF becoming too small). The lower concentration limit is set by the number of detected independent events becoming too small (and can be pushed using beam scanning [11,13], as done here). For comparison’s sake, we therefore chose [*C*] ~ 1 molecule μm^−2^ (*N* ~ 0.5) for the studied sample, which is at the upper limit of what is acceptable for SPT and in the lower range of what is acceptable for FCS.

A second important point of comparison between methods is their capacity at separating mobile from immobile particles and, more generally, to deal with complex heterogeneous samples. In the studied complex membrane, both confocal images and TIRF movies clearly showed the presence of immobile diffraction-limited fluorescent particles. Those might have corresponded to fluorescent probes inserted in immobile lipid domains, interacting with the solid substrate, or to part of unfused liposomes. In order to obtain the diffusion coefficient of the mobile fluorescent probes in the membrane, these stationary particles needed to be eliminated. Due to its single-particle nature, SPT presents an easy way to identify immobile particles, just by considering the average step size of each individual particle (as was done here). On the other hand, FCS has been traditionally considered as unable to deal with immobile fractions. Our study, however, raises two interesting points in that regard. Firstly, we note that although SPT returns single-particle data, the short length of the tracks obtained in most cases (see Figure 4) in fact precludes obtaining real single-particle information; averages must be taken over many particles in order to obtain a diffusion coefficient with reasonable precision. Secondly, there are ways of correcting the FCS data that allow removing the contribution of immobile particles. For example, this can be done for RICS by subtracting an average image intensity [16,48]. In this study, we implemented a relatively simple way of removing immobile particle contributions, by selecting small areas of the collected images and checking that the average line cross-correlation function for that area (between the in focus image and that acquired immediately above) had a zero amplitude. This method was successful; however, it requires a sample that does display areas free of immobile particles, something that might not always be possible. It also entails a bias in how the smaller areas are chosen and a loss of information, since the presence of immobile particles is then ignored. Finally, using smaller areas means that the obtained line auto-correlation functions have more noise, and the result of their analysis is less precise. Overall, however, the perceived superiority of SPT at dealing with complex heterogeneous samples depends very much on the possibility to obtain long tracks for single particles, which can happen only under certain conditions, the quantum yield and photostability of the fluorophore being especially important.

Finally, and most importantly, the two chosen techniques here notably differ in the range of diffusion coefficients that they can measure. Let us consider the values of the diffusion coefficient measured here for DiD in the mitochondria-like membrane using FCS and SPT and, for each of these techniques, different methods of analysis. These values are summarized in Table 1. We first see that the measured diffusion coefficients are reasonable for a lipid-like probe in a fluid solid-supported lipid bilayer at room temperature, usually found to be between 1 and 5 μm^2^ · s^−1^ [22,23,45]. In addition, in a previous study where SPT was used to study the same lipid bilayer, a diffusion coefficient of *D* = 2.2 μm^2^ · s^−1^ has already been reported [10]. We then note that, given a certain experimental method, different analyses return slightly different values of the diffusion coefficient, but this difference is not significant. On the other hand, the values of the diffusion coefficient measured using FCS and SPT are slightly, but statistically different. For example, *D* = (3.4 ± 0.4) μm^2^ · s^−1^ for line-scanning FCS using full images, and *D* = (2.4 ± 0.3) μm^2^ · s^−1^ for SPT using the MSD of individual tracks. We note that discrepancies in measured values of *D* could in principle be due to deviation from simple diffusion, for example anomalous diffusion [49,50], or binding events between periods of simple diffusion [51]. However, we argue here that the slight difference in the value of *D* measured by both methods might in this particular case be due to their limited dynamic range and to the fact that for this system (for which *D* is in the higher range of what can be measured by SPT and in the lower range of what can be measured by FCS), SPT tends to underestimate *D*, while FCS tends to overestimate it.

**Table 1:** Measured diffusion coefficients for DiD in the mitochondria-like membrane. Errors are the standard error of the mean (SEM), except for the value of *D* extracted from the MSD of the combined track, which was calculated by adjusting the fitting window, as explained in the text, and for that extracted from the distribution of displacements, in which case, the error is the standard deviation of the values obtained for different lag times.

Experimental Method	Analysis Method	*D* (μm^2^ · s^−1^)
Line-scanning FCS	Full images	3.4 ± 0.4
	Cropped images	3.2 ± 0.4
SPT	MSD of individual tracks	2.4 ± 0.3
	MSD of combined track	2.2 ± 0.4
	Distribution of displacements	1.6 ± 0.2

The limitation with SPT comes from which types of particles can actually be located in an image, given a certain acquisition time *τ_a_*, as mentioned for example in [52]. In order to be properly detected, a particle needs to remain localized within a surface area roughly corresponding to the point spread function. If not, it would appear as a blur rather than a single diffraction-limited particle and would not be recognized by the SPT algorithm, or at least not localized accurately. This means that *D_max_* ~ *πω*^2^/(4*τ_a_*) ~ 5 μm^2^ · s^−1^ (for *τ_a_* = 30 ms). This value can be increased, allowing one to capture faster motions, by decreasing the acquisition time, as shown for example in [26]. However, this is not always possible. In the sample studied here, for example, the signal-to-noise ratio was around 2 to 3, *i.e.*, at the lower limit of what is necessary for single particle detection. Decreasing acquisition time was therefore not an option. The signal-to-noise could in principle be increased by increasing the excitation power; however, this is done at the risk of increasing photobleaching, which, in turn, limits the capacity of detecting long particle trajectories. For the sample studied here, the excitation power had been chosen to achieve both an acceptable signal-to-noise ratio and an acceptable level of photobleaching. It could not have been increased without detrimental effects. Thus, in the end, what eventually limits the capacity of SPT to capture fast diffusive motions is the quality of the fluorescent probe (in particular, its quantum yield and its resistance to photobleaching) and the efficiency of the light collection system (including noise rejection).

There are several limitations to consider with line-scanning FCS, as discussed for example in [9]. The first limitation is that the time-resolution of the ACF, set by the pixel dwell time *δ* (which determines at which lag time the first point in the ACF after that obtained for *τ* = 0 will be), needs to be significantly smaller than the characteristic diffusion time $\tau_{D}=\omega_{0}^{2}/\left(4D\right)$. Therefore, only diffusion coefficients $D\ll \omega_{0}^{2}/\left(4\delta \right)$ are accessible with this technique or, reversing the argument, one needs to choose the pixel dwell time, such that $\delta \ll \omega_{0}^{2}/\left(4D\right)$, in order to capture the dynamics of the particles. Here, assuming *D* ~ 5 μm^2^ · s^−1^, *i.e.*, on the higher end of what is expected for probes in fluid solid-supported membranes, we see that we need *δ* ≪ 6 ms, justifying our choice of *δ* in the range of 0 to 3 ms. A second limitation is that the characteristic scan time *τ_S_* = *ω*_0_*δ*/*d* should not be much smaller than the characteristic diffusion time *τ_D_*. It has been speculated in the past that one strictly needs *τ_S_* > *τ_D_*/2 (corresponding to the case when the main decay in the ACF is linked to the value of *τ_D_* and, thus, when it becomes very easy to extract this value) [9]. However, our present study shows that *τ_D_* can still be extracted from the ACF as long as *τ_S_* > *τ_D_*/3 (see Figure 2). Our success at extracting *τ_D_* in these conditions is probably at least partially due to the fact that the fit was done including the value of *G*(0), which our algorithm successfully calculated, something that is not often done in other studies. Line-scanning FCS thus allows measuring diffusion coefficients *D* > *ω*_0_*d*/(12*δ*) ~ 1 μm^2^ · s^−1^ in the conditions of our experiment.

We note that the scanning conditions are important. As stated above, we need *δ* ≪ *τ_D_*, and the choice of the pixel-to-pixel distance *d* will influence the range of available *δ*. Choosing a smaller *d* (which reduces the distance between pixels and, therefore, the scanning speed; in the limit *d* → 0 the experiment becomes a single-point FCS experiment) in principle allows one to access smaller diffusion coefficients. However, it also reduces the range of concentrations that are accessible (here, because we worked in the lower concentration range for FCS, scanning was helpful in obtaining ACFs with good statistics, which is why *d* = 100 nm was chosen). Furthermore, eventually, one becomes limited by photobleaching if *τ_D_* is less than the photobleaching time, *i.e.*, the average time necessary to photobleach the fluorophore under the considered excitation conditions, *τ_P_* = *hc*/*σ_P_λ*Φ. The photobleaching cross-section of DiD at *λ* = 637 nm is *σ_P_* = 1.3 × 10^−13^ μm^2^ [37]. At the excitation flux used here, $\Phi =I/\pi \omega_{0}^{2}$ = 6.5 kW·cm^−2^ (with *I* = 25 μW), we can estimate *τ_P_* ~ 40 m. Therefore, in the conditions of our experiments, even if we had chosen a smaller *d*, a hard limit for *D* would be $D_{\text{min}}=\omega_{0}^{2}/\left(4\tau_{P}\right)$ ~ 0.8 μm^2^ · s^−1^. This value will of course depend on the excitation power used and the quality of the dye; however, those do not vary immensely between studies.

Our study underscores the strengths and weaknesses of both line-scanning FCS and SPT. The former is a good technique for reasonably homogeneous samples for a wide range of fluorophore concentrations (including very low [*C*] when using fast scanning and high concentrations not accessible to SPT), but only for particles with relatively fast diffusion (*D* > 1 μm^2^ · s^−1^ for standard dyes and imaging conditions). The latter is restricted to very low fluorophore concentrations and particles with relatively slow diffusion (*D* < 5 μm^2^ · s^−1^ for standard dyes and imaging conditions), but remains necessary in the case of highly heterogeneous samples. Other complementary fluorescence techniques are available to study membrane diffusion. Foremost amongst those is FRAP, an ensemble technique, which typically requires concentrations higher than those used in FCS and only allows studying molecules with relatively slow diffusion coefficients (*D* < 1 μm^2^ · s^−1^ for raster-scanning FRAP experiments as implemented on commercial confocal microscopes [9]). Of note, the issue of limited dynamic range to be avoided by using non-fluorescence techniques, such as pulsed field gradient NMR, which gives access to mean-squared displacements by ensemble averages (rather than time averages for SPT). This method has been used very successfully to study lipid diffusion [53,54]. It cannot, however, be used *in vivo*.

Placing this in the context of membrane diffusion, we see that SPT just about allows studying the diffusion of lipid probes in solid-supported lipid bilayers. It is not adequate for anything diffusing faster, which includes lipid probes in giant unilamellar vesicles (where lipid diffusion is often faster than 2 μm^2^ · s^−1^ [23]) and any protein that is not inserted in the lipid bilayer, but bound electrostatically to the membrane (as some forms of the protein Bid, for example [10]). FCS, on the other hand, is ideal for such fast diffusing particles and just about allows studying the diffusion of lipid probes in solid-supported lipid bilayers. It might not always be appropriate for the diffusion of lipids in cell membranes, which are often more viscous than artificial membranes [28]. Neither is it an appropriate technique to study membrane-embedded proteins or proteins partially inserted in the lipid bilayer, as those diffuse significantly slower than the surrounding lipids [55] and tend to exist in several different conformations, then making SPT the technique of choice to resolve this heterogeneity.

## 4. Methods

### 4.1. Supported Lipid Bilayer Preparation

As a model system, we used a solid-supported lipid bilayer with a composition reflecting the average composition of the inner and outer mitochondrial membranes of yeast [56]. The lipid components (all purchased dissolved in chloroform from Avanti Polar Lipids) were phosphatidylcholine (egg PC) at 48 mol%, phosphatidylethanolamine (egg PE) at 28 mol%, phosphatidylinositol (liver PI) at 10 mol%, phosphatidylserine (DOPS) at 10 mol% and cardiolipin (TOCL) at 4 mol%. In addition, 0.0025 wt% of the lipophilic dye 1,1′-dioctadecyl-3,3,3′,3′-tetramethylindocarbocyanine, 4-chlorobenzenesulfonate (DiD-C_18_, from Life Technologies, dissolved in methanol) was added.

The solid-supported lipid bilayer was created mostly as previously described [10]. Briefly, all components (with a total lipid mass of ~ 1 mg), including DiD, were mixed together and placed in a borosilicate glass tube. The solvents (chloroform and methanol) were removed using first a steady stream of helium and then incubation at a pressure of 20 mHg for 3h, until a dry lipid film had formed on the sides of the tube. The lipid film was then redissolved in assay buffer (10 mM HEPES pH 7, 200 mM KCl, 1 mM MgCl2, and 0.2 mM EDTA) and submitted to a freeze-thaw process to render the liposomes unilamellar. This was followed by extrusion through a polycarbonate membrane with a 100 nm pore size. Approximately 0.5 mL of the liposome solution was then deposited on a freshly-cleaved mica sheet glued on a 40 mm diameter 170 μm-thick glass coverslips placed in an FCS2 sealed perfusion chamber (Bioptechs, Butler, PA). The chamber was incubated at 38 °C for 2 h to support liposome fusion. In the final step, the sample was washed with assay buffer (~5 mL, one drop per second) to remove excess lipids.

### 4.2. Line-Scanning Fluorescence Correlation Spectroscopy

#### 4.2.1. Confocal Imaging

Confocal image stacks of solid-supported lipid bilayers were acquired on an Insight Research Spectrometer (Evotec Technologies, Hamburg, Germany). The excitation source was a POLAR 635-25 continuous-wave solid-state laser (Coherent, Santa Clara, CA, USA). The excitation beam (power *I* = 25 μW) was set to overfill the back aperture of the 100× water-immersion objective (numerical aperture of 1.30) used for imaging. In conjunction with the 40-μm confocal pinhole used in the detection path, this resulted in a confocal detection volume with a ~350-nm radius and a ~3-μm height. The emitted fluorescent light was detected by avalanche photo-diodes (SPCM-CD3017, Perkin-Elmer Optoelectronics, Wellesley, MA, USA). All images were 100 × 100 pixels and 10 × 10 μm in size, *i.e.*, the pixel size (distance between two consecutive pixels) was *d* = 100 nm. The value of the pixel dwell time, *δ*, varied. Typically stacks of 5 to 10 images were acquired, with a distance of 0.5 μm to 1 μm between each image in the stack. This ensured that, in at least one image in the stack, the lipid bilayer was in focus.

#### 4.2.2. Generation and Analysis of Line Autocorrelation Functions

In each image stack, the image with the highest average fluorescence was selected, as the most likely to have captured the lipid bilayer in focus. Fluorescence from the lipid bilayer could also usually be detected in adjacent images. An average line autocorrelation function (line ACF, *G*(*τ*)) was then computed for the brightest image, using a dedicated plugin written for ImageJ, as described previously [36]. This plugin contains a correction to remove shot-noise contribution in the correlation function. Because shot noise is a random process, fluctuations in intensity due to shot noise are uncorrelated in time. In other words, the value of the shot noise in a pixel cannot help predict the value of the shot noise in the next pixel. Thus, shot noise does not contribute to *G*(*τ*), unless *τ* = 0. On the other hand, $G\left(0\right)=\delta i/\langle i\rangle =\sigma_{N}^{2}+\sigma_{i}^{2}$, where $\sigma_{N}^{2}=1/\langle N\rangle$ is the variance in the number of particles, *N*, present in the confocal detection volume, while $\sigma_{i}^{2}=1/\langle i\rangle$ is the variance in the number of photons (or shot noise), *i*, detected in a pixel. The plugin we implemented calculates $G\left(\tau \right)=\langle \delta i\left(t\right)\delta i\left(t+\tau \right)\rangle /{\langle i\rangle }^{2}-<i>\delta_{k}\left(\tau \right)$, where *δ_k_*(*τ*) is the Kronecker delta function. In the previous formula, *i* always represents the background subtracted intensity, where the background coming from the single-photon detectors is subtracted first. This is very important when working at a low concentration, *i.e.*, when 〈*i*〉 is on the same order of magnitude as the background. With the shot noise correction, we have *G*(0) = 1/〈*N*〉. For this work, the line cross-correlation function (line CCF, *G_X_*(*τ*)) between the brightest image and the next image in the stack was also computed, in order to detect the eventual presence of immobile particles.

For a single fluorescent species diffusing in two dimensions in the lipid bilayer (with a diffusion coefficient *D*), one expects the line ACF to take the form [37]:

(1) G(τ)=1N11+ττDexp-ττS21+ττD

where *N* is the average number of fluorescent particles found in the confocal detection volume (*N* is therefore directly related to the fluorescent particle concentration in the lipid bilayer), $\tau_{D}=\omega_{0}^{2}/4D$ is the characteristic diffusion time and *τ_S_* = *ω*_0_*δ*/*d* is the characteristic scanning time.

For immobile particles, *i.e.*, *τ_D_* → ∞, we get:

(2) G(τ)=1Nimmobileexp-ττS2

*N*_immobile_ is the average number of immobile particles (*i.e.*, fluorescent particles with *τ_D_* larger than the image acquisition time) present in the confocal detection volume.

In the case of two consecutive images in the stack, one expects correlations only if immobile fluorescent particles are present and, therefore, visible in both images. When such particles exist, the line CCF between the two images should have the same form as Equation (2). In the case when no immobile particles are present, *G_X_*(*τ*) = 0.

When both mobile and immobile particles are present, we get:

(3) G(τ)=1Nf×11+ττDexp-ττS21+ττD+(1-f)×exp-ττS2

where *f* is an effective fraction of mobile particles (it is the actual fraction only if the brightness of mobile and immobile particles is the same).

### 4.3. Single-Particle Tracking

#### 4.3.1. Total Internal Reflection Fluorescence Microscopy

The imaging setup is built on an objective-based TIRF Nikon Ti eclipse inverted microscope (Nikon Instruments Inc., Melville, NY, USA). A Nikon CFI60 oil immersion objective with 100× magnification and numerical aperture (NA) of 1.49 was used. The excitation light source was a solid state laser emitting at 647 nm. With the appropriate excitation and emission filters in place, the fluorescence emission intensity and position are detected by an Andor iXon 897 EMCCD camera (Andor Technology Ltd., Belfast, U.K.). Time-lapse movies were acquired at a frame rate of ~33 fps (acquisition time of *τ_a_* = 30 ms per frame) and a field of view of 512 × 512 pixels with pixel sizes of 160 nm × 160 nm.

#### 4.3.2. Generation and Analysis of Single-Particle Tracks

To analyse the movies, a program was written in Python under the use of the packages numpy, scipy and PIL, using a standard single-particle detection algorithm [57,58,59]. Each frame was first processed with a Gaussian and a top-hat filter. Local maxima were detected, and a threshold was applied to create a binary map, providing an initial estimate of the particle positions. With these positions as input, the unfiltered image was then fitted by two-dimensional Gaussians via least squares optimization. Detections were checked via the width and eccentricity of the fitted Gaussian functions and discarded if values exceeded appropriate thresholds. The acquired localizations give the fluorophore position with an approximate accuracy of ~30 nm [58]. The tracks were created by linking individual positions from consecutive frames. Each link was associated with a cost, representing the travelled distance of the particle in a frame interval. The individual particle tracks were generated by minimizing the global cost for all links.

Three different approaches were then taken to determine the average diffusion coefficient. The first two were based on the analysis of the mean-squared displacement (MSD), which for diffusive behaviour is linear in time:

(4) $$\langle {\left(\Delta r\right)}^{2}\rangle \left(\tau \right)=4D\tau$$

The first approach consisted of appending all tracks to create a master trajectory, for which the MSD was calculated and fitted using Equation (4). In the second approach, the MSD was calculated for each individual track and fitted with Equation (4), resulting in a value of the diffusion coefficient for each particle, which could then be averaged.

The third approach was based on the analysis of the distribution of displacements. For a simple diffusion process, in each dimension the probability density for a displacement Δ*x* to occur after a lag time *τ* is given by:

(5) $$P\left(\Delta x,\tau \right)=\frac{1}{\sqrt{4\pi D\tau }}\exp\left[-\frac{\Delta x^{2}}{4D\tau }\right]$$

The distribution of displacements obtained for all tracks were binned and fitted via Equation (5) for different lag times, *τ*, in order to obtain a value of the diffusion coefficient.

## 5. Conclusion

In conclusion, we observe that SPT and FCS have in fact a very small overlap in terms of dynamic range (*D* ~ 1 to 5 μm^2^ · s^−1^ in standard imaging conditions), which is right in the middle of the range of diffusion coefficients found in lipid membranes, and where the diffusion coefficient of the studied sample lies. FCS and SPT should thus be thought of as complementary methods for studying membrane dynamics. Whereas FCS allows detecting fast diffusing particles such as lipid molecules in very fluid membranes (such as those of giant unilamellar liposomes) and loosely-bound proteins hovering above the membrane, SPT allows studying lipid molecules in more viscous membranes (such as cell membranes) and proteins inserted into membranes.
